# Platinum, or Continuous Gum Work

**Published:** 1856-01

**Authors:** W. B. Roberts

**Affiliations:** New York


### ARTICLE VII.

### *Platinum, or Continuous Gum Work.*

Dr. Blandy:

Dear Sir—Your favor of the 27th of December, was duly received, and in reply to your inquiries relative to the meeting of dentists, held at the Dental College in Philadelphia, on the evening of the 8th of December, I would state that the discussion upon that occasion respecting the platinum or continuous gum work was very limited. The object of the meeting was not to discuss its merits or demerits, but simply to place before the dentists of Philadelphia, specimens of work which carried with them more convincing arguments than weeks or months of discussion could have effected. Two years of experience in this style of work, with results that have far surpassed my most sanguine expectations, led me to believe that it was yet in its infancy. And learning from Dr. John Allen and others, that but very little of this work was being done in that city, (a city, too, that can boast of the best block workers in the world,) it occurred to me that this place should participate, with others, in bringing to greater perfection the work over which Dr. John Allen has spent years of anxiety and toil, and which now bids fair to amply repay him, (if not pecuniarily,) in the consciousness of being instrumental in placing before the profession a work unsurpassed in natural appearance to any other method yet known.

I accordingly conversed with Dr. J. L. Clark, (who was at this time staying in New York, and whose professional abilities I highly esteem,) with the view of bringing the subject more especially before the profession. The result of which was an interview with some dentists of Philadelphia, among whom was Dr. Elisha Townsend. The meeting of the 8th was determined upon, and well attended by the dentists of Philadelphia. Dr. Townsend stated the object of the meeting, and introduced Dr.

Clark and myself. Dr. Clark stated that his experience in the work warranted him in recommending it to the careful investigation of the profession, notwithstanding the great barrier (patent right,) which seems to retard its progress.

I was also called upon to exhibit several specimens I had with me, which I am happy to say gave, as far as I could learn, much greater satisfaction than we had anticipated. Upon the points of beauty and natural appearance, there appeared to exist but one opinion, but in regard to durability and the greater weight which must always exceed the old style, there was the same opinion that exists in every mind that is unaccustomed to it. Upon these points I stated, what I now state in this letter, that a set of teeth, properly put up and with a perfect fit, upon the continuous gum plan, exceeds all other modes in point of strength, cleanliness, beauty and natural appearance.

Yours, with respect,

W. B. Roberts.

New York, *Jan. 5th,* 1856.

